# Global and Regional Prevalence of Carpal Tunnel Syndrome: A Meta‐Analysis Based on a Systematic Review

**DOI:** 10.1002/msc.70024

**Published:** 2024-12-13

**Authors:** T. Gebrye, E. Jeans, G. Yeowell, C. Mbada, F. Fatoye

**Affiliations:** ^1^ Department of Health Professions Manchester Metropolitan University Manchester UK; ^2^ Orthopaedics North Shore Hospital Auckland New Zealand; ^3^ Lifestyle Diseases Faculty of Health Sciences North‐West University Potchefstroom South Africa

**Keywords:** carpal tunnel syndrome, musculoskeletal disorder, prevalence, systematic review

## Abstract

**Background:**

Carpal tunnel syndrome (CTS) is a considerable concern, impacting individual health and socio‐economic factors. A systematic review and meta‐analysis of CTS prevalence would offer valuable insights for healthcare planning, improving outcomes and reducing the burden on affected individuals.

**Methods:**

In line with the Preferred Reporting Items for Systematic Reviews and Meta‐Analyses (PRISMA) guidelines, a meta‐analysis was conducted to estimate the prevalence of CTS. Medline, CINAHL, AMED, Scopus, and Web of Science databases were searched for studies published from 1 January 2012 to 10 October 2024. The pooled prevalence rates were determined using a random effects model.

**Results:**

The search yielded 548 initial findings, 103 duplicate records were eliminated, and only 31 of these papers were deemed relevant for inclusion in this review. The prevalence estimates were sourced from 15 different countries, including the United States (*n* = 8), Saudi Arabia (*n* = 5), Ethiopia (*n* = 3), Turkey (*n* = 2), Iran (*n* = 2) and Brazil (*n* = 2), among others. Each of the following countries contributed one study: China, France, Germany, India, Kuwait, the United Kingdom, Korea, the Netherlands, and Sweden. In total, the included studies analysed 5,311,785 individuals, revealing a prevalence of CTS ranging from 0.003 to 0.743. The random‐effects meta‐analysis yielded an overall prevalence estimate of 0.144, with a 95% confidence interval (CI) of 0.067–0.282, based on 30 studies.

**Conclusion:**

The prevalence estimates for CTS are notably high, highlighting the need for effective surgical management strategies. Developing and implementing these interventions is crucial to enhancing health outcomes for individuals affected by CTS.

## 1 Introduction

Carpal tunnel syndrome (CTS) is one of the most prevalent musculoskeletal disorders (MSDs), it is a chronic focal compressive neuropathy (Genova et al. 2020) and carries a significant socio‐economic burden (Dale et al. 2013). Risk factors for CTS include repetitive hand movements in typing‐related occupations, specific anatomical and physiological traits, age, gender, and pregnancy (Nowak et al. 2023). Previous epidemiological studies have also identified several conditions associated with CTS, which can be categorised into constitutional, hormonal, and musculoskeletal factors. Constitutional risk factors encompass obesity and smoking (Nathan, Meadows, and Istvan 2002). Hormonal risk factors include diabetes, hypothyroidism, the use of combined oral contraceptives, hormonal replacement therapy, and corticosteroid use in the absence of inflammatory arthritis (Ferry et al. 2000). Idiopathic CTS is the most common diagnosis for patients experiencing these symptoms, typically confirmed through electrophysiological testing and clinical assessments (Wipperman and Goerl 2016).

The management of CTS involves both surgical and non‐surgical approaches. Surgical treatment is typically reserved for patients with severe symptoms, as noted by Karjalanen et al. (2022). Non‐surgical interventions, including oral steroids, wrist splints, ultrasound therapy, laser therapy, and local steroid injections, have been shown to have varying effectiveness for mild to moderate symptoms, with results differing in both the short and long term (Panathoop, Saengsuwan, and Vichiansiri 2023). Conservative strategies such as splinting, corticosteroid injections, and physical therapy are often preferred initially (Carlson et al. 2010). Recent research emphasises a multidisciplinary approach that includes ergonomic adjustments and lifestyle modifications to reduce recurrence and enhance long‐term recovery outcomes (Rotaru‐Zavaleanu et al. 2024).

Healthcare providers typically compile a comprehensive case history that highlights the characteristic signs and symptoms of CTS to aid in its diagnosis (Moosazadeh et al. 2018). Given the increasing demands on limited healthcare resources, data on the prevalence of CTS are essential for effective planning of medical services. Evidence indicates that the economic impact of CTS goes beyond direct costs, encompassing significant indirect costs as well (Foley, Silverstein, and Polissar 2007). The prevalence of CTS directly influences the number of workdays lost, resulting in financial losses for both patients and society (US Bureau of Labor Statistics 2015). Therefore, optimising patient outcomes while reducing healthcare costs and alleviating the economic burden of missed work due to CTS is becoming increasingly vital.

Epidemiological data on the prevalence of CTS are crucial for identifying risk factors and improving our understanding of the disease's progression within populations (Genova et al. 2020; Foroozanfar et al. 2016; Saha, Chant, and Mcgrath 2008). The prevalence estimates for CTS have shown considerable variation across different studies and countries. These discrepancies can be attributed to demographic differences in the studied populations as well as variations in the methodologies employed (Saha, Chant, and Mcgrath 2008). While systematic reviews and meta‐analyses are crucial in providing insights into the prevalence of CTS, they are often limited by factors such as geographic focus, population‐specific findings, variations in diagnostic methods, and selective reporting (Kostares et al. 2023; Palmer, Harris, and Coggon 2007; Moharrami, Charsouei, and Dehkharghani 2023; Chenna et al. 2023). This means that findings from these reviews may not fully capture the global burden of CTS, and further studies encompassing diverse populations and settings would be needed to gain a more accurate understanding of its prevalence across the world. Additionally, recent prevalence data can help identify emerging trends, promote health equity, and provide a foundation for proactive, evidence‐based health policies that improve population health and well‐being. Therefore, the objective of this review was to systematically assess the global prevalence of CTS.

## 2 Methods

This systematic review adhered to the guidelines outlined by the Preferred Reporting Items for Systematic Reviews and Meta‐Analyses (PRISMA) (Liberati et al. 2009). A protocol for the review was prospectively registered with PROSPERO and is accessible at PROSPERO (https://www.crd.york.ac.uk/prospero/display CRD: CRD42022342029).

### 2.1 Data Sources and Search Strategy

Search strategies for identifying studies on the prevalence of CTS were developed through discussions among the reviewers. The databases searched included Medline, CINAHL, AMED, Web of Science, and Scopus, using specific terms related to CTS and focussing on prevalence studies. The search terms, such as ‘prevalence’, ‘epidemiology’, and ‘carpal tunnel syndrome’, were combined using conjunctions such as ‘AND’ and ‘OR’. Additionally, a manual search of the reference sections of the included studies was conducted to identify further relevant research. The search process was carried out by one author (TG) and cross‐checked by a second author (CM) to minimise bias in the selection and exclusion of studies.

### 2.2 Inclusion and Exclusion Criteria

We set a date limit of January 2012 for study inclusion, as a systematic review conducted several years ago may not reflect recent changes in the prevalence of CTS, especially in response to new workplace practices, ergonomics interventions, or increasing rates of screen time and sedentary behaviour. We included studies that reported the prevalence of CTS across all age groups, utilising cross‐sectional, retrospective, and prospective designs. Eligible studies had to be published in English and available in full text. We excluded conference proceedings, review articles, studies published before 2012, articles in press, abstracts, editorials, guidelines and recommendations, as well as non‐English studies.

### 2.3 Study Selection and Quality Assessment

After removing duplicates, two reviewers (TG & CM) independently screened the titles, abstracts, and full‐text articles. Any discrepancies were resolved through discussion and consensus among the reviewers (FF, GY & EJ). A quality assessment was conducted for each study using the criteria established by Hoy et al. (2012). Each study was assigned a score ranging from 0 to 10 based on its adherence to 10 criteria concerning external and internal validity. The responses to these criteria were categorised as either ‘yes’ or ‘no’. Studies scoring 9 or 10 ‘yes’ answers were deemed to have a low risk of bias, those with scores of 7 or 8 were classified as having a moderate risk of bias, while studies with scores of 6 or lower were considered to have a high risk of bias.

### 2.4 Data Extraction

Data extraction was conducted using an Excel spreadsheet designed to capture several domains, including study reference, location, data source, methodology, age, number of individuals with CTS, total number surveyed, and prevalence. Additionally, if available, the breakdown of prevalence by sociodemographic categories (e.g., age, sex) was recorded.

All extracted data were independently assessed by two reviewers (TG & CM). In cases where discrepancies arose during the data extraction process, a third reviewer (FF) was brought in to reassess the relevant study. Any differences in findings were discussed among the reviewers until a consensus was reached.

### 2.5 Data Synthesis

Descriptive analyses were performed to summarise the demographic data for each study. Using Comprehensive Meta‐analysis software (Biostat, Inc., New Jersey, USA) version 3 for Windows, we calculated the pooled prevalence of carpal tunnel syndrome (CTS) along with 95% confidence intervals (CIs). A random effects model was employed to account for the variation within individual studies as well as the variance between studies (Stroup et al. 2000). This model is particularly suitable when significant heterogeneity in prevalence exists across studies. Heterogeneity was evaluated using the *I* ^2^ statistic, with thresholds of ≥25%, ≥50%, and ≥75% indicating low, moderate, and high heterogeneity, respectively (Higgins et al. 2003).

## 3 Results

**FIGURE 1:**
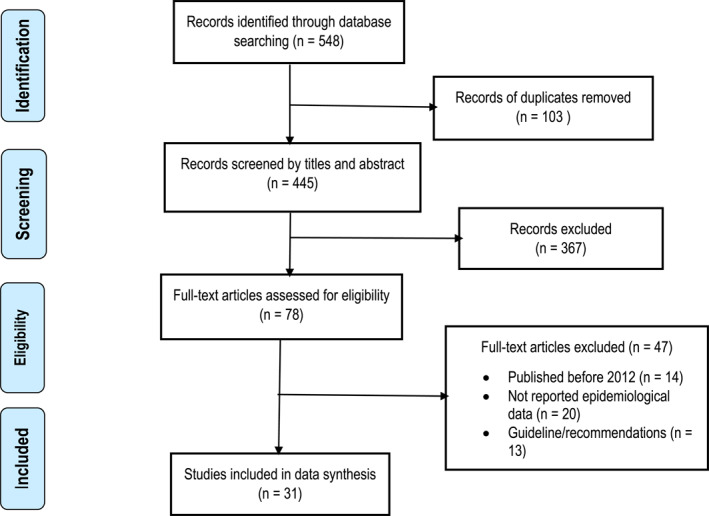
Flow diagram of publications included and excluded in the review.

The search strategy identified 548 references published between January 2012 and September 2024. After removing 103 duplicates, we proceeded with 445 references for screening based on titles and abstracts. Following a full‐text review, 31 studies were ultimately included in this review (see Figure 1). Out of these, 30 studies were deemed eligible for meta‐analysis. Among the included studies, 23 were classified as having a low risk of bias, while the remaining 8 were assessed as having a moderate risk of bias (Table 1).

**TABLE 1:** Summary of study characteristics and overall prevalence of carpal tunnel syndrome.

Authors, Year	Location	Data source	Age, years	Number with CTS	Number surveyed	Prevalence	Risk of bias
Ahamed et al. (2015)	Saudi Arabia	Cross‐sectional study	All age groups	57	225 participants	25.30%	Low
Akbar et al. (2014)	Germany	Retrospective	Range: 32–72	35	56 paraplegic and wheelchair dependent	63%	Low
Alhusain et al. (2019)	Saudi Arabia	Cross‐sectional	>30	67	223 dentists	30.5% [95% CI 0.25 to 0.36]	Low
AlHussain et al. (2023)	Saudi Arabia	Cross‐sectional study	Mean (SD), 40.8 (8.0)	45	490 teachers	9.10%	Moderate
Bicha et al. (2024)	Ethiopia	Cross‐sectional study	18 and 70	39	333 construction industry workers	11.7% (AOR: 95% CI: 8.1–15.3)	Low
Bekele et al. (2022)	Ethiopia	Cross‐sectional	>18	11	353 diabetic patients	3.1%	Moderate
Burton et al. (2018)	UK	Primary care database	>18	12,530 person‐year	473,094 person years	26.02 per 10,000 person‐years [95% CI 23.45 to 36.72]	Low
					0.026		
Cartwright et al. (2012)	USA	Prospective cohort study	≥18	25	287 Latino poultry processing workers	8.70%	Moderate
Demissie et al. (2023)	Ethiopia	Cross‐sectional study	Mean (SD), 29.2 (9.1)	49	422 computer user bankers	11.7%	Low
Demiryurek and Gündoğdu (2018)	Turkey	Questionnaire	Mean: 31.1 ± 7.3	81	110 women hairdressers	The prevalence of CTS among hairdressers 74.3%	Low
Feng et al. (2021)	China	Cross‐sectional survey	Range: 17–49	93	969 Chinese office workers	9.60%	Low
Hemaxi, Murugan, and Limbasiya (2019)	India	Cross‐sectional study	NR	16	105 adult obese individuals	15%	Moderate
Jung et al. (2016)	South Korea	Cross‐sectional study	>30	194	377 subjects (174 men and 203 women)	The prevalence of CTS 51.5%	Low
Kadow et al. (2018)	USA	Cross‐sectional study	≥18	812	56,641 patients in the orthopaedic department	1.40%	Low
Khosrawi and Maghrouri (2012)	Iran	Cross‐sectional	Range: 17–41	19	100 pregnant women	19%	Moderate
Khired et al. (2024)	Saudi Arabia	Cross‐sectional survey	Mean (SD), 43.3 (6.5)	27	336 teachers	8.00%	Moderate
Kurtul and Mazican (2023)	Turkey	Cross‐sectional study	Mean (SD), 37.4 (8.3)	112	151 hospital office workers	74.10%	Low
Luckhaupt et al. (2013)	USA	Cross‐sectional study	≥25	1174	17,524 adults	6.7% (6.3%–7.2%)	Low
Mirghani et al. (2024)	Saudi Arabia	Cross‐sectional study	Range: 18–25	13	384 (general population)	3.40%	Moderate
Meems et al. (2015)	Netherlands	Prospective longitudinal cohort study	<35	219	639 Dutch pregnant women	34%	Low
Meirelles et al. (2020)	Brazil	Cross‐sectional study	>14	15	72 athletes	20%	Moderate
K. M. Musolin and Ramsey (2017)	USA	Cross‐sectional survey	Range: 20–70	64	191 workers	The prevalence of CTS 34%	Low
K. Musolin et al. (2014)	USA	Cross‐sectional survey	Range: 19–73	126	301 plant production employees	42%	Low
Manes (2012)	USA	Cross‐sectional study	Mean: 48	15	50 long‐term bikers	30% of participants, in the left hand in 12%, and bilaterally in 8%	Moderate
Oliveira et al. (2019)	Brazil	Cross‐sectional study	NR	111	482 women	23.03%	Low
Patil et al. (2012)	USA	Cross‐sectional study	Range: 26–44	11	66 dairy workers	16.60%	Moderate
Pendleton et al. (2024)	USA	Cross‐sectional study	Between the ages of 41–60 (range: 41–78)	44	101 neurosurgical spine surgeons	43.60%	Low
Raman et al. (2012)	Kuwait	Cross‐sectional	≥20	88	470 office workers	18.70%	Low
Ridderström et al. (2020)	Sweden	Questionnaire (patient history & clinical examination)	Range: 25–86	8	23 individuals known for heterozygous mutation	#Hereditary sensory, 35%	Moderate
						#Autonomic neuropathy group, 52%	
Roquelaure et al. (2017)	France	HD, SI & RD	Range: 20–59	5459	18,34,093 inhabitants	3.08 (95% CI 2.11–4.06) per 1000 person‐years	Low
Yazdanpanah et al. (2012)	Iran	Cross‐sectional	Range: 25–50	51	1508 pregnant women	3.40%	Low

Abbreviations: AOR = adjusted odds ratio; CI = confidence interval; CTS = carpal tunnel syndrome; HD = hospital discharge records; NR = not reported; RD = region's work‐related diseases surveillance programme among the region's salaried workers; SD = standard deviation; SI = social insurance; USA = United States of America.

### 3.1 Characteristics of the Included Studies

**FIGURE 2:**
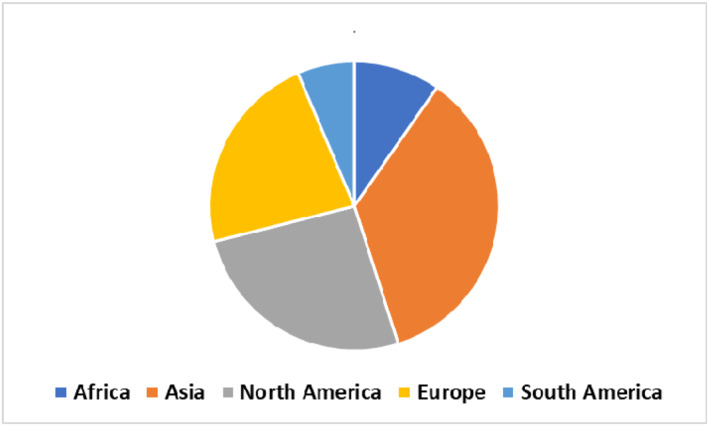
Proportion of the included studies by regions (*N* = 31).

The characteristics of the included studies are summarised in Table 1. All studies were published after January 2012, with prevalence estimates derived from 15 countries, including the United States (Cartwright et al. 2012; Kadow et al. 2018; Luckhaupt et al. 2013; K. M. Musolin and Ramsey 2017; K. Musolin et al. 2014; Manes 2012; Patil et al. 2012; Pendleton et al. 2024), Brazil (Meirelles et al. 2020; Oliveira et al. 2019), Saudi Arabia (Alhusain et al. 2019; Ahamed et al. 2015; Mirghani et al. 2024; AlHussain et al. 2023; Khired et al. 2024), Iran (Khosrawi and Maghrouri 2012; Yazdanpanah et al. 2012), Ethiopia (Bekele et al. 2022; Bicha et al. 2024; Demissie et al. 2023), Turkey (Demiryurek and Gündoğdu 2018; Kurtul and Mazican 2023), and one study each from China, France (Roquelaure et al. 2017), Germany (Akbar et al. 2014), India (Hemaxi, Murugan, and Limbasiya 2019), Kuwait (Raman et al. 2012), the United Kingdom (Burton et al. 2018), South Korea (Jung et al. 2016), the Netherlands (Meems et al. 2015), and Sweden (Ridderström et al. 2020). Most of the included studies were from Asia (35.5%) followed by North America (26%) (Figure 2).

The included studies collectively enrolled a total of 5,311,785 participants, with sample sizes ranging from 11 in the USA (Patil et al. 2012) to 1,834,093 in France (Roquelaure et al. 2017). Except for four studies (Cartwright et al. 2012; Demiryurek and Gündoğdu 2018; Hemaxi, Murugan, and Limbasiya 2019; Oliveira et al. 2019), all studies reported the ages of the participants. Notably, one study indicated that participants included individuals from all age groups (Ahamed et al. 2015).

### 3.2 Overall Prevalence

**FIGURE 3:**
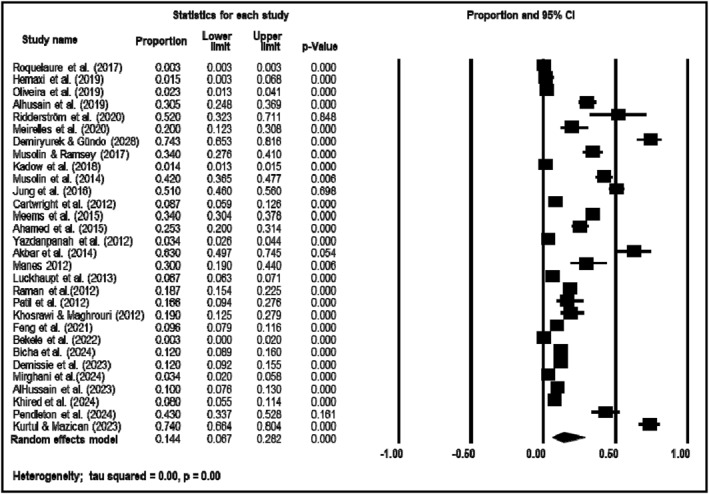
Meta‐analysis of CTS overall prevalence using a random effects model.

The analysis of CTS prevalence included 30 studies with a total of 1,917,082 participants (median: 317; range: 23 to 18,340). The overall pooled prevalence estimates from the meta‐analysis are shown in Figure 3. The combined estimate of CTS prevalence across all studies during the entire period was 0.144 (95% CI: 0.067–0.282), with individual estimates varying from 0.003 (95% CI: 0.00–0.02) to 0.743 (95% CI: 0.653–0.816).

### 3.3 Prevalence of CTS in High Income Countries and LMICs

**FIGURE 4:**
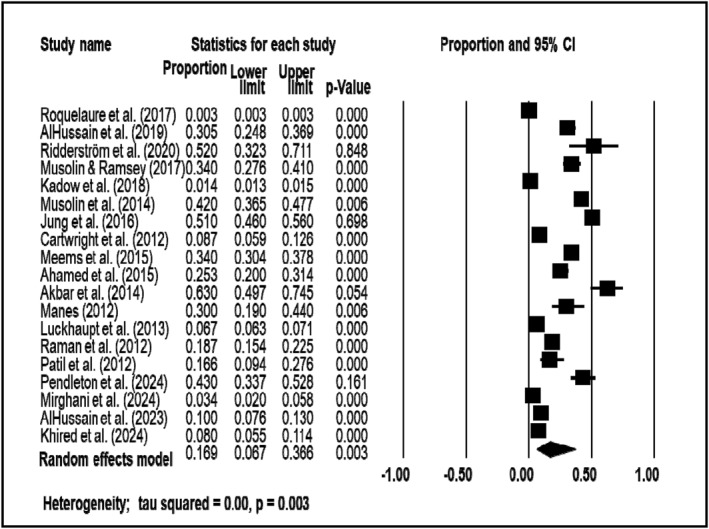
Meta‐analysis of CTS in high income countries.

**FIGURE 5:**
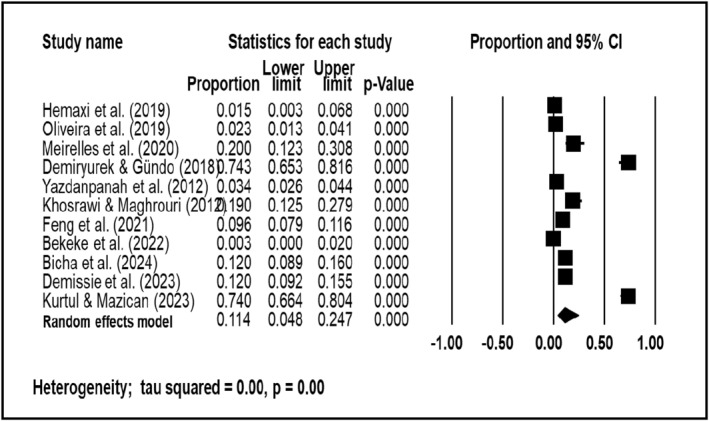
Meta‐analysis of CTS in LMICs.

The review included a total of 19 studies on CTS prevalence in high‐income countries and 11 studies in low‐ and middle‐income countries (LMICs). The studies in high‐income countries involved a sample population of 1,912,477 participants (median: 301; range: 23 to 1,834,093), while the studies in LMICs comprised 4605 participants (median: 333; range: 72 to 1508). Sub‐analysis by income level revealed notable differences in prevalence. The random effects analysis showed that the prevalence of CTS in high‐income countries was 0.169 (95% CI: 0.067–0.366) (see Figure 4), whereas in low‐ and middle‐income countries, the prevalence was 0.114 (95% CI: 0.048–0.247) (see Figure 5).

### 3.4 Regional Prevalence of CTS

**FIGURE 6:**
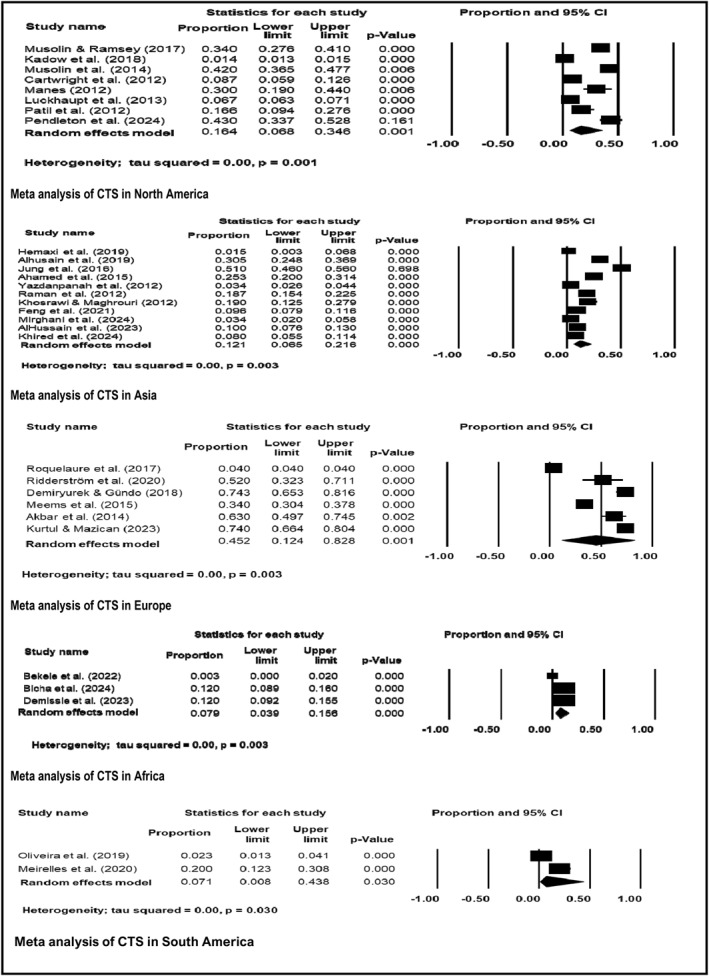
Meta‐analysis of CTS by continents.

The pooled prevalence of CTS in various regions is presented in Figure 6. The prevalence of CTS was 0.164 (95% CI: 0.068–0.346) in North America (eight studies), 0.121 (95% CI: 0.065–0.216) in Asia (eleven studies), 0.452 (95% CI: 0.124–0.828) in Europe (six studies), 0.079 (95% CI: 0.039–0.156) in Africa (three studies), and 0.071 (95% CI: 0.008–0.438) in South America (two studies).

## 4 Discussion

The systematic review and meta‐analysis of studies on the prevalence of CTS employed subgroup analyses to investigate how income levels—low‐middle versus high‐income countries—may influence prevalence rates. The quality assessment of the included studies revealed that 75% had a low risk of bias, while 25% exhibited a moderate risk of bias. Our findings indicate that approximately 14.4% of the global population experiences CTS. Furthermore, the review showed that the prevalence of CTS was 11.4% in low‐ and middle‐income countries and 16.9% in high‐income countries.

Our results were compared with previous meta‐analyses on CTS prevalence in the United States (Genova et al. 2020) and Iran (Moosazadeh et al. 2018). The overall prevalence of CTS in our study was consistent with findings from the USA (7.8%) and Iran (17%). The lower prevalence reported in the USA may be attributed to the study population, which primarily included individuals employed in settings where some or all workers engaged in hand‐intensive activities. In contrast, the higher prevalence observed in our analysis could be due to the inclusion of more recent studies from a diverse range of countries, providing a broader perspective on CTS prevalence globally.

Our findings indicate that the overall prevalence of CTS is higher in high‐income countries compared to low‐ and middle‐income countries. This observation aligns with existing literature, which suggests that increased strain and repetitive movements in occupational settings contribute to this trend. Many Western nations have seen a rise in work‐related musculoskeletal disorders (Punnett and Wegnman 2004). For instance, a study in the fish processing industry reported CTS prevalence rates as high as 73% among workers (Jenkins et al. 2012). This underscores the substantial impact of CTS in high‐income countries, highlighting it as a significant public health concern for authorities.

The meta‐analysis by Moosazadeh et al. (2018) reported a prevalence of 17.5% for CTS across various populations in Iran, revealing a 3% difference compared to our study. One potential reason for this discrepancy could be the varying levels of experience among physicians in diagnosing CTS. Additionally, patients may come from diverse socioeconomic and educational backgrounds, which can make it challenging for them to articulate their symptoms effectively (Mody et al. 2009).

The findings of the current review indicate that the reported prevalence of CTS varies by Gross National Income (GNI) of countries. This review also highlights the challenges associated with conducting epidemiological evaluations across diverse populations and regions. For instance, one study (Burton et al. 2018) did not provide the specific data required for inclusion in the meta‐analysis. Additionally, our limitation to English‐language papers excluded many relevant studies published in other languages. Furthermore, inconsistencies in age group distribution among some papers prevented us from extracting data for all age groups, making it impossible to standardise the results for age and sex.

## 5 Conclusion

The prevalence estimates for CTS are notably high globally, with significant rates observed in both high‐income and low‐to middle‐income countries. These findings hold significant implications for public health authorities, as they can guide the formulation of strategies for early diagnosis and effective treatment of individuals with symptomatic CTS.

## Author Contributions

T.G., C.M. and F.F. participated in the design of the review, the literature search, the extraction of data and the methodological appraisal of studies. T.G., C.M., G.Y., E.J. and F.F. participated in the conceptualisation, design, literature search, writing of the manuscript, and interpreted the results of the work. All authors approved the final version of the manuscript.

## Ethics Statement

The authors have nothing to report.

## Conflicts of Interest

The authors declare no conflicts of interest.

## Data Availability

The authors have nothing to report.
